# Bioethanol Production from Stalk Residues of Chiquere and Gebabe Varieties of Sweet Sorghum

**DOI:** 10.1155/2021/6696254

**Published:** 2021-02-18

**Authors:** Nitin Mahendra Chauhan, Sunil Tulshiram Hajare, Buzuayehu Mamo, Abreham Assefa Madebo

**Affiliations:** Department of Biology, College of Natural and Computational Sciences, Dilla University, Dilla 419, SNNPR, Ethiopia

## Abstract

Bioethanol produced from renewable resource has potential to solve environmental pollution and to satisfy the need of demand and supply. It favours the use of nonfood lignocellulosic materials. Ethanol produced from plant materials can sustain the economy by reducing cost of imported petroleum, emitting neutral CO_2_. Moreover, it enhances the economy by providing value added market opportunities for transportation and agricultural sector. Therefore, the objective of the study was to investigate bioethanol production from stalk residues of Chiquere and Gebabe varieties of sweet sorghum collected from West Arsi Zone, Ethiopia. Response surface methods with a three factor (inoculum size, pH, and dilution rate) with triplicate run by using the Box–Behnken method was referred. The experiment employed dilute acid hydrolysis, because it is an easy and productive process by treating the stalks with 4% of sulfuric acid for effective hydrolysis of substrate. Finally, the fermentation was carried out at 30°C for 72 hours on a shaker at 180 rpm by using *Saccharomyces cerevisiae*. The significance of the result was evaluated by using ANOVA, where *P* values <0.05 were considered statistically significant. In the process, maximum yield of ethanol was obtained at an inoculum size of 5% (22.40%), pH level of 4.0 (21%), and dilution rate at 10 ml (21.46%). Very low yeast inoculum size and dilution factor have positive effect on the yield of ethanol, whereas very high dilution rate produced negative impact on ethanol production. FTIR spectroscopy peaks associated with O-H, C-O, and C-H stretching vibrations confirmed the presence of ethanol obtained from sweet sorghum stalks. The results of our study indicated that, being available in bulky amounts and nonedible material, sweet sorghum stalks can serve as potential feedstock for bioethanol production in developing countries such as Ethiopia.

## 1. Introduction

Bioethanol is an alternative source of energy to be known in the value adding performances, and therefore, it significantly contributes to the reduction of crude oil consumption, mitigating deleterious impacts on environmental factors associated with green gas emission and air pollution. Because of burning of petroleum-based fuels leads to rise of CO_2_ level in the environment, bioethanol from waste can serve as an alternative source of energy in the future. Consequently, it is an ongoing interest to find out a renewable and environmentally friendly source of energy for our industrial economies and consumer societies [1].

Ethanol fermentation from carbohydrates is considered as probably one of the oldest processes known to man. Today, it is an important potential alternative source of liquid fuels for the transport sector. Typically, lignocellulose to ethanol processes consist of at least four steps. These are pretreatments to enhance biomass digestibility, hydrolysis of cellulose to sugar monomers, fermentation of sugars to ethanol, and recovery of ethanol by distillation from process stream [2].

*Saccharomyces cerevisiae* is the common yeast which is normally preferred for ethanol production because of more ethanol productivity, high ethanol tolerance, and ability of fermenting varieties of sugars. However, there are some challenges in yeast fermentation which inhibits ethanol production such as high temperature, high ethanol concentration, sugar concentration, pH, fermentation time, agitation rate, dilution rate, inoculum size, and the ability to ferment pentose sugars [3, 4].

Bioethanol produced from feedstock is classified into two generations. The first-generation bioethanols are the most widely produced from corn, wheat, oil, maize, cassava tubers, and other starchy and sugar-based feedstock sources for bioethanol production [5]. However, security concerns regarding use of food crops for bioethanol production favour the use of nonfood lignocellulosic materials, which comprise inexpensive, nonedible, and abundant biomass in the form of agricultural and forestry residues [6]. Besides, first-generation bioethanol has some limitations due to the competition with food production changes in the land use, and high water requirements. Thus, great attention has been paid to second-generation bioethanols that are produced from lignocellulose feedstock such as nonfood crops, straw, grass, sawdust, sorghum stalk, and wood chips. In addition, second-generation ethanol has much higher potential for greenhouse gas emissions reduction than first-generation ethanol [7].

Nowadays, burning of fossil fuels has led significant increase in the global CO_2_ concentrations, while it leads to the global warming with extreme changes in climate and weather, worldwide. Besides, fossil fuels are not going to last forever because depletion of the world's petroleum resources is inevitable. Again, in the future, the cost of fossil fuel might be very expensive for demand. Thus, alternative energy sources need to be renewable, sustainable, efficient, cost-effective, and safe. Among renewable fuels, ethanol due to its inherent characteristics such as less toxic to the microbes, nature friendly, and low boiling point and due to the presence of oxygen, it provides a cleaner and more efficient fuel after burning [8]. When it is used in vehicles such as cars, ethanol reduces the amount of carbon dioxide and comparable energy content for primary fuel candidate for near or long-term application [9].

Ethanol produced from plant materials is a sustainable economy by reducing cost of imported petroleum, emitting neutral CO_2_, and it boosts economy providing value added market opportunities for transportation and agricultural sector. The use of grain for bioethanol production may cause inflation of cost of these grains leading to food insecurity. To alleviate such problems, searching nonedible residues and high production of lignocellulose biomass were envisaged as representing an alternative source of energy, more sustainable feed stock source, and low cost [10].

Sorghum plants are rich in cellulose and lignocellulose which can be used as a good substrate for ethanol production. Sweet sorghum bagasse has 14–21% of lignin, 18–27% of hemicellulose, and 34.35 % of cellulose content [11]. This lignocellulosic biomass chemical structure causes key challenges to development and commercialization for ethanol because lignin surrounds the hemicellulose and cellulose. Pretreatment of biomass is necessary to make the biomass susceptible to microorganisms and improve economic value and ethanol yield. All the fermentable sugars in sweet sorghum are converted to ethanol [12]. Sweet sorghum is an indigenous African plant belonging to the family Poaceae and scientifically known as *Sorghum bicolor* (L.) Moench [13]. Sweet sorghum is also characterized by experience of self-pollination, tolerance to biotic and abiotic stress such as low water requirement, and resistance to salty, alkaline soils and temperature [14,15], and adaptability to a wide range of environmental conditions could prove an ideal crop for ethanol production [16].

Previous studies have been investigated on Brawley, Sart, and Rio varieties of sweet sorghum for bioethanol production [17, 18]. However, in Ethiopia, very little attention has been given to utilization of the sorghum variety of stalk residues for ethanol production. Therefore, this work is the first approach to determine the role of Chiquere and Gebabe varieties in ethanol production. The results of the proposed work are highlighted and discussed below.

## 2. Materials and Methods

### 2.1. Description of Study Area

**Figure 1:**
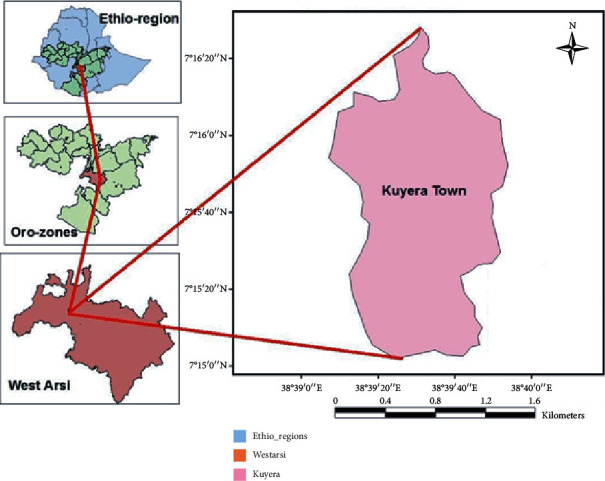
Map of the study area.

The study was conducted towards southeastern direction approximately 250 km far away from Addis Ababa, in Kuyera district, West Arsi Zone, Ethiopia. It is approximately located between 38° 4' and 8° 32' E and 7° 3' and 2° 20' N (Figure 1) and extends over a latitudinal range from 1557 to 1650 m with the total area of 449.6 hectares. Andosol soil type covers about 52.2% while nitosols cover the remaining 47.8%. The main crops produced for consumption purpose and for income generation were potatoes, maize, sorghum (finger millet, Chiquere, Gebabe, and white seed color varieties), wheat, barley, and teff [19].

### 2.2. Sample Collection and Preparation

Gebabe and Chiquere varieties were selected for bioethanol production, from other varieties based on the local availability of stalks and have no more economic value after drying rather than burning in the farm land. Both variety stalk residues were collected from the Kuyera district in 2019. The stalks were selected by simple probability sampling techniques from farm land after harvesting of sorghum grain randomly after dry. The leaves were stripped off from the stalks by hand for animal feed. The collected sweet sorghum variety stalks were transported to Department of Biology and Chemistry Research Lab, Dilla University, Dilla, Ethiopia. The stalk residues were washed by running water to remove dirt (if present) and dried by the sun for two days followed by oven drying at 60°C for 72 hours [20]. Stalks were broken down into small pieces with the aid of mortar and pestle which was suitable to ground. Then, the grounded powder was broken by coffee grinding electronic machine up to the size of 2 mm and sieved for experimental purpose. The prepared samples were packed in the bottle for laboratory analysis. The experiments of bioethanol production from stalk residues of Chiquere and Gebabe varieties of sweet sorghum were carried out at the School of Chemical and Bioengineering Laboratory, Addis Ababa University, Addis Ababa, Ethiopia.

### 2.3. Experimental Design

60 g of the substrate was used in batch pretreatment and hydrolysis process in 600 ml dilute sulfuric acid (H_2_SO_4_), to enhance the rate of conversion from polysaccharides into monosaccharides. For this study, diluted H_2_SO_4_ (2% for pretreatment and 4% for hydrolysis process) was mixed with the substrate at 1 : 10 solid-to-liquid ratio to be treated inside an autoclave at 121°C for 50 minutes and hydrolysed at 150°C for 1 hour. 1 N of sodium hydroxide and hydrochloric acid were used to adjust the pH [21]. Batch fermentation of the experiment was designed by a factorial design with three factors (inoculum size, pH, and dilution factor) and three replications for each treatment of the two varieties and one positive control group for both varieties. This fermentation was carried out using baker's yeast *Saccharomyces cerevisiae*. The negative control was yeast mixed with water, while positive control was yeast served with glucose [22]. After adjusting the pH at 3, 4, and 5 and dilution for 10 ml, 20 ml, and 30 ml and inoculum size of 5%, 10%, and 15%, the impact of those variables on the ethanol yield was evaluated. Fermentation media was incubated at a temperature of 30°C and stired at 180 rpm for 72 hours [3]. The independent variables were pH, inoculum size, and dilution. The dependent variable was ethanol yield.

### 2.4. Characterization of Stalk Residues of Chiquere and Gebabe Varieties

#### 2.4.1. Determination of Moisture Content

The empty evaporated dishes were weighed on an electronic balance before adding the sample. Then, 3 g of the sample powder was placed in an empty evaporated dish. The weighed samples were placed in an oven at 105°C for 3 hours to vaporize the water. Then, the evaporated dish was transferred into a desiccator and allowed to cool before determining its weight. The dried samples were weighted three times, and the mean of weighted sample was determined [23]. Moisture content was calculated using the following formula:

(1) $$\begin{array}{c} \text{moisture content }\left(\%\right)=\left(\frac{\left(W_{1}-W_{2}\right)}{W_{1}}\right)\times 100, \end{array}$$

where *W*_1_ = weight of the evaporated dish with sample before drying (3 g); *W*_2_ = weight of the evaporated dish and sample after drying.

#### 2.4.2. Volatile Matter Content

The empty crucibles were weighed on an electronic balance before adding the sample. Then, 3 g of the sample was added in it. The sample and the crucible were placed in a muffle furnace for 30 min at 600°C. The crucible was removed from the furnace and placed in a desiccator to cool, and then the weight was reweighed until constant weight was obtained [23]. The volatile content was determined as follows:

(2) $$\begin{array}{c} \text{volatile content }\left(\%\right)=\left(\frac{\left(W_{1}-W_{2}\right)}{W_{1}}\right)\times 100, \end{array}$$

where *W*_1_ = original weight of the sample (3 g); *W*_2_ = weight of the sample after cooling.

#### 2.4.3. Extractives Analysis

6 g of the oven dried sample was weighed and placed into Trimble, which is plugged with filter paper and placed in a Soxhlet extraction tube. An exhaustive ethanol extraction was completed in 6 hours using the Soxhlet method. The residue was then dried in the oven at 105°C for 4 hours at a constant weight and cooled at room temperature in a desiccator and reweighed. The weighed difference between the varieties of sweet sorghum sample before and after leaching was the amount of the extractives [23]. The extractive contents in the raw sweet sorghum variety sample were calculated as follows:

(3) $$\begin{array}{c} W_{1}\text{ dry basis weight }\left(\%\right)=\left(\frac{\left(W_{1}-W_{2}\right)}{W_{1}}\right)\times 100 \end{array}$$

where *W*_1_ = weight of the origin sample (5 g); *W*_2_ = weight after drying.

#### 2.4.4. Determination of Lignin

1g of extractive-free sample was placed in the conical flask and 12 ml of 72% sulfuric acid was added. 100 mL of distilled water was added to the mixture. After distilled water addition, the mixture was boiled for 2 hours and cooled at room temperature. The insoluble material (lignin) was filtered by using Whatman paper (no. 42). The lignin was washed with distilled water until neutralized with H_2_SO_4_ and then oven dried at 105°C for 10 hours at a constant weight and cooled down in a desiccator and weighed [7, 24]. Lignin content was calculated as follows:

(4) $$\begin{array}{c} \text{lignin content }\left(\%\right)=\left(\frac{\left(W_{1}-W_{2}\right)}{W_{1}}\right)\times 100, \end{array}$$

where *W*_1_ = oven dried sample (1 g); *W*_2_ = extracted residues.

#### 2.4.5. Analysis of Hemicellulose

2 g of the extractive-free dried sample was placed in a conical flask and 150 mL of NaOH solution (20 g/L) was added to the sample. The mixture was boiled for 3 hours, filtered, and washed with distilled water to remove the NaOH solution. The residue was then dried in an oven for 5 hours at 105°C at a constant weight, cooled at room temperature in a desiccator, and reweighed (*W*_2_). The difference between the sample weight before and after treatment was the hemicellulose content [23]. The hemicellulose content sweet sorghum variety was calculated as follows:

(5) $$\begin{array}{c} \text{hemicellulose content }\left(\%\right)=\left(\frac{\left(W_{1}-W_{2}\right)}{W_{1}}\right)\times 100, \end{array}$$

where *W*_1_ = weight of the sample before drying (2 g)*; W*_2_ = weight of the sample after drying.

#### 2.4.6. Determination Content of Cellulose

The cellulose content was calculated in different ways, assuming that extractive hemicellulose and lignin are the only components of the entire sweet sorghum variety stalk residues [23] as follows:

(6) $$\begin{array}{c} \text{total biomass}=W_{\text{C}}+W_{\text{E}}+W_{\text{H}}+W_{\text{L}},\\ W_{\text{C}}=100-W_{\text{C}}+W_{\text{E}}+W_{\text{L}}, \end{array}$$

where *W*_C_, *W*_H_, *W*_L_, and *W*_E_ are cellulose, hemicellulose, lignin, and extractive content, respectively.

#### 2.4.7. Determination of Reducing Sugar

The total reducing sugar contents of Chiquere and Gebabe varieties were determined through phenol sulfuric acid method by taking anhydrous glucose as the standard. To prepare stock solution, 0.5 g of glucose was dissolved into 100 ml of distilled water in a volumetric flask. Six test tubes were prepared for standard solution; one test tube for blank and the five test tubes were for glucose standard of 20 mg/ml, 40 mg/ml, 60 mg/ml, 80 mg/ml, and 100 mg/ml. 1 ml of phenol solution (5 g/100 mL) and 1 ml of the sample were added into the test tubes. Then, 5 ml of 96% concentrated sulfuric acid was added rapidly to produce a good mixture. The test tubes were allowed to stand for 10 min to develop [13]. The absorbance of the sample was measured at 490 nm by using a UV-visible spectrophotometer using glucose as the standard [25]. A graph was plotted against the standard to get the total sugar.

### 2.5. Methods of Bioethanol Production

#### 2.5.1. Pretreatment

Dilute sulfuric acid 2% was used for the treated solution inside an autoclave and heated at a temperature of 121°C, for 30 minutes by 1 : 10 solid-to-liquid ratio pretreated inside an autoclave at 140°C for reaction time of 50 minutes [26, 27]. After cooling and filtered through a filter paper (Whatman no. 42), the filtrated contents were preserved in another conical flask and were kept in a refrigerator at 4°C for further use. The pretreated solid residues were washed twice by distilled water to remove sulfuric acid from it and dried before keeping for hydrolysis purpose. Stalk powder was fed as batches, and every batch contains 60 g of screened stalks powder with a ratio of 1 : 10 (w/v) sample to the water [12].

#### 2.5.2. Hydrolysis

Acid hydrolysis procedure was started by adding 4% diluted sulfuric acid to the nonsoluble component from pretreatment steps of 60 g. The stalk residues were hydrolysed inside an autoclave and heated at the temperature of 150°C, for 90 minutes. After cooling, the liquid was separated from solid particles by filtering through a filter paper (Whatman no. 42). After separation, the solid parts were washed with distilled water two times. Finally, pretreated and hydrolysate filtered solutions were mixed together and preserved in a refrigerator [28].

#### 2.5.3. Preparation of Media

*Saccharomyces cerevisiae* was collected from Ethiopian Health and Nutrition Research Institute (EHNRI) and transported to a laboratory in ice box and culture in yeast extract broth. The conical flask was placed on a shaking incubator adjusted at 180 rpm and incubated at 30°C for 24 hours [29].

#### 2.5.4. Preparation of Fermentation Medium

Fermentation media were prepared by placing 100 ml of pretreated and hydrolysed solution into 250 ml flasks. Pretreated and hydrolysed solution was mixed before addition of any microorganism to the prepared samples. The pH of these samples was adjusted between 3.0 and 5.0 before shaking the substrate by using a digital pH meter. The reactor and all the equipment that were used for fermentation purposes were autoclaved at the temperature of 121°C for 15 minutes. After it was cooled in the hood, activated yeast was added into the fermentation medium. Batch types of fermentation were conducted in incubators preset at 180 rpm for 72 hours at 30°C.

#### 2.5.5. Effect of Different Factors on Ethanol Production

The effect of yeast inoculum size on ethanol yield was determined by varying the yeast inoculum size to 5%, 10%, and 15% [3]. Also, the effect of pH on the ethanol yield was determined by varying the pH level of the fermentation broth for 3.0, 4.0 and 5.0 [3]. Finally, the effect of dilution rate on ethanol production during fermentation was determined by varying the addition of 10 ml, 20 ml, and 30 ml water into the prepared solution [30].

### 2.6. Distillation

The fermented 100 ml of fermented broth was centrifuged at 1000 rpm to remove the unused starch and yeast cell for 5 minutes. The fermented media were distilled through batch distillation at a temperature of 78°C by using the rotary evaporator for 2-3 hrs [31].

### 2.7. Estimation of Ethanol Concentration

The amount of ethanol produced by fermentation was estimated by potassium dichromate method [14, 25]. The experiment was performed by dissolving 1 g of potassium dichromate into 27 ml of 98% of sulfuric acid solution. Five clean and dry test tubes were taken containing 00 ml, 0.2 ml, 0.4 ml, 0.6 ml, 0.8 ml, and 1.00 ml of known ethanol concentration, while the sixth test tube was used as blank. Then, 3 ml of prepared chromic acid solution was added to each test tube followed by placing in the water bath at 60°C for 10 minutes. In the end, 1 ml of Rochelle salt solution (40%) was added to stabilize the color. The absorbance was measured at 600 nm using a UV-VIS spectrophotometer.

### 2.8. Fourier Transform Infrared Spectroscopy Test

The identification of functional group of ethanol was carried out by FTIR spectroscopy analysis in the Department of Chemistry, College of Natural and Computational Science, Addis Ababa University, Addis Ababa, Ethiopia. The samples were examined and the result was checked by comparing with standard ethanol graph by referring standard methodology [24].

### 2.9. Data Analysis

Various Design Expert 7 software data analysis tools were used to determine the effect of yeast inoculum size, pH, and dilution rate. The response variable was ethanol yield. The significance of result was calculated by analysis of variance (ANOVA). *P* value <0.05 was considered statistically significant.

## 3. Results

### 3.1. Characterization of Stalk Residuals of Chiquere and Gebabe Varieties

As observed from Table 1, moisture contents of stalk residues of Chiquere and Gebabe varieties were 4.33% and 6.67%, respectively, while the volatile matter in stalk residues of Chiquere and Gebabe varieties was 48% and 42.67%, respectively. The stalk residuals of Chiquere variety had high volatile matter content than stalk residuals of Gebabe variety. Chiquere variety of sweet sorghum stalk powder has high extractives in the form of 2%, lignin 22%, and hemicellulose 37% and 41.5% of cellulose contents as compared to Gebabe variety stalk powder which contains 1.8% of extractives, lignin 20%, hemicellulose 34% and cellulose 39.2% (Table 1).

### 3.2. Determination of Reducing Sugar

The total sugar content found in stalk residues of Chiquere variety was 6.67%, 9.12%, 12.5%, 15.83 %, and 22.5 %; on the other hand, in stalk residues of Gebabe variety, sugar content was 5.42%, 7.5%, 10.83%, 13.33%, and 21.25% (Table 2).

### 3.3. Estimation of Ethanol Concentration

The concentrations of unknown ethanol were determined by measuring the absorbance of know ethanol concentrations at 600 nm (Table 3). The minimum and maximum range of ethanol produced from Chiquere variety was 32% to 40.88%, while, in Gebabe variety, it was found to be 31.73% to 38.33%, whereas positive control produced 32.8% to 45.67% of ethanol. High amount of ethanol was produced from glucose as compared to both varieties of sweet sorghum.

### 3.4. Experimental Analysis of Ethanol

To determine whether the quadratic model is significant, it was crucial to perform analysis of variance (ANOVA). The result of ANOVA analysis for ethanol yield in Chiquere and Gebabe varieties is highlighted in Table 4. The model *F*-value of 73.59 for Chiquere variety and 120.3 for Gebabe variety implies the model is significant. Values of Prob > *F* less than 0.0500 indicate model terms are significant. In this case, A, C, AB, AC, BC, A2, B^2^, and C^2^ are found to be significant models in both varieties. The lack of fit *F*-value of 12.42 for Chiquere variety and 0.28 for Gebabe one imply that the lack of fit is not significant which means the model fits well (Table 4). The details of model adequacy measures of Chiquere and Gebabe varieties of ethanol yield of sweet sorghum are shown in Table 5. The Pred R-Squared of 0.8473 when compared to the Adj R-Squared of 0.9761 for Chiquere variety is reasonably accepted. Similar trend was seen in Gebabe variety also. A ratio greater than 4 is desirable. A ratio of 26.388 for Chiquere one and 39.937 for Gebabe variety indicates an adequate signal. As such, this model can be used to navigate the design space. In the end, the designed experimental data presented in Table 6 indicate the 95% CI high and low values of each model term for both varieties of sweet sorghum studied.

### 3.5. Diagnostic Plot for Ethanol Production

**Figure 2:**
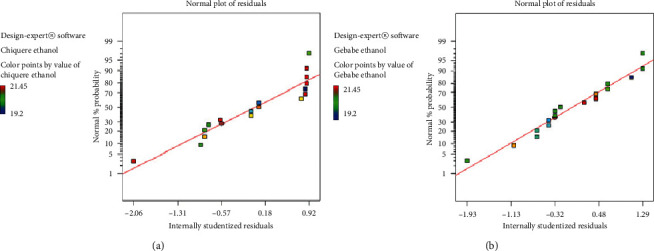
Normal plot of residuals for ethanol production from Chiquere and Gebabe varieties of sweet sorghum. (a) Chiquere variety; (b) Gebabe variety.

**Figure 3:**
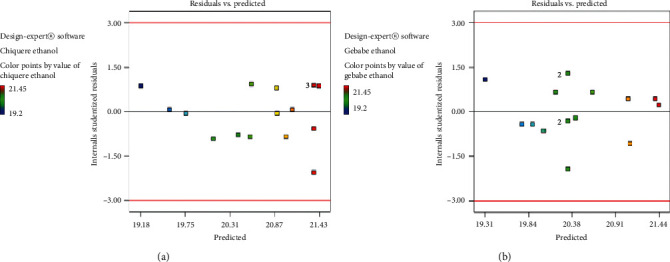
Plot of residuals versus model predicted values for ethanol production from Chiquere and Gebabe varieties of sweet sorghum. (a) Chiquere variety; (b) Gebabe variety.

Diagnostic plot represented in Figure 2 for ethanol yield from Chiquere and Gebabe variety indicates that the residuals followed a normal distribution. In case of this experiment, the points indicated in the plot were fit to a straight line. This showed that the quadratic polynomial model satisfies the assumptions analysis of variance. There is no severe indication of abnormality as well as pointing of possible outliers. The points are coded by color to the level of response and they represent going from cool blue for lowest values to hot red for the highest. The plots of residuals versus predicted (Figure 3) also confirmed the assumption of the analysis of variance (ANOVA). In the analysis of variance, it is usually more effective and straight forward to do this with the residuals. Thus, Figure 3 showed resembles as a straight line.

### 3.6. Contour Plot of the Effect of Different Parameters on Ethanol Yield

#### 3.6.1. Inoculum Size versus pH

**Figure 4:**
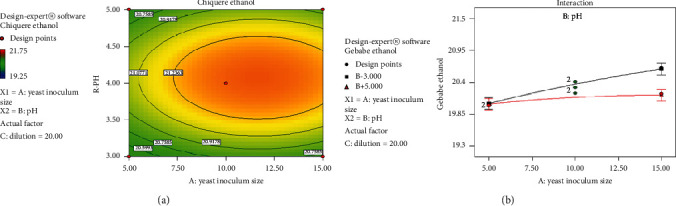
Contour plot for the effect of inoculum size and pH on ethanol yield. (a) Chiquere variety; (b) Gebabe variety.

The contour plot concerning effects of inoculum size and pH on the Chiquere and Gebabe varieties of ethanol yield, when the dilution factor is at the center, are represented in Figure 4. From the contour plot, it is predicted that response of ethanol yield is a function of inoculum size and pH as there is a color change on the graph and the response variable increasing from green to yellow color. The graph suggests operating at the center point where the response variable is at a maximum. Operating in the yellow color region is good to have high amount of ethanol yield. This indicates that there is high interaction between inoculum size and pH. As shown in Figure 4(b), black and red lines indicate low and high level of parameters, respectively.

#### 3.6.2. Inoculum Size versus Dilution Factor

**Figure 5:**
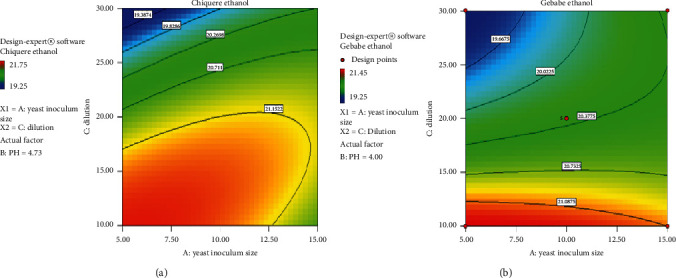
Contour plot for the effect of inoculum size and dilution factor on ethanol yield. (a) Chiquere variety; (b) Gebabe variety.

The contour plot in terms of effects of inoculum size and dilution interaction on the Chiquere and Gebabe varieties of ethanol yield, when the pH is at the center, are shown in Figure 5. From the contour plot, it is estimated that the response of ethanol yield is a function of inoculum size and pH as there is color change on the graph and the response variable decreases from the initial to edge. The difference is indicated by different colors: red to yellow followed by green and blue color from maximum variable response to minimum response. The graph suggests operating at the initial point where the response variable is at maximum. Operating in the red color region is good to have high amount of ethanol yield. There is high interaction between inoculum size and dilution at minimum variable level for stalk residuals of both varieties. In addition to this, as the level of variable increases, the amount of ethanol yield was decreased. However, as color indicated, the amount of Chiquere variety ethanol yield was greater than the amount of Gebabe variety ethanol yield.

#### 3.6.3. pH versus Dilution Rate

**Figure 6:**
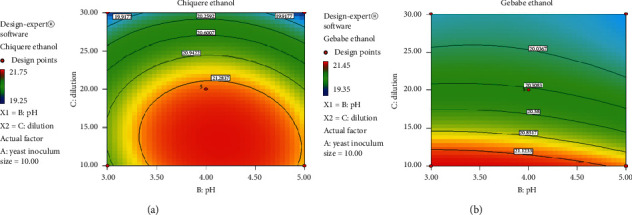
Contour plot for the effect of pH and dilution factor on ethanol yield. (a) Chiquere variety; (b) Gebabe variety.

The contour plot of effects of pH and dilution interaction on the Chiquere and Gebabe varieties of ethanol yield, when the yeast inoculum size was at the center point, are highlighted in Figure 6. There was color change on the plot and the response variable increases from green to yellow to red color. The graph suggests the operation around the middle point where the response variable shows maximum amount of ethanol yield. Particularly, the maximum amount of ethanol yield was found around pH 4.0 levels for Chiquere variety, whereas, in Gebabe variety, it was found around the initial point even though Gebabe variety ethanol yield was less than Chiquere variety. Operating in the red region is good to have high amount of ethanol yield.

### 3.7. Factors Affecting Ethanol Production during Fermentation

#### 3.7.1. Inoculum Size

The highest ethanol yield after 72 hours of fermentation through stalk residues of Chiquere variety was 22.40%, 21.57%, and 19.87%, while, in Gebabe variety, it was noted as 22.17%, 21.78%, and 19.88% at 5%, 10%, and 15% of yeast inoculum size, respectively. Therefore, 5% yeast inoculum size resulted in higher ethanol yield as compared to 10% and 15% (Table 7).

#### 3.7.2. pH

Ethanol yield after 72 hours of fermentation for stalk residues of Chiquere variety was 19.94%, 21%, and 20.92%, while, for those in Gebabe variety, it was noted as 19.76%, 21.71%, and 20.53% at 3, 4, and, 5 pH respectively. Therefore, at 4 pH level, higher ethanol yield was produced as compared to 3 and 5 pH levels for both varieties (Table 7).

#### 3.7.3. Dilution Rate

Ethanol content after 72 hours of fermentation via stalks of Chiquere variety was 21.4%, 20.11%, and 19.55%, while, in Gebabe variety, it was estimated as 21.78%, 21.29%, and 19.86% for 10 ml, 20 ml, and 30 ml dilution rate, respectively. Therefore, dilution rate of 10 ml produced higher ethanol yield as compared to 20 ml and 30 ml dilution rate for both varieties (Table 7).

### 3.8. Fourier Transform Infrared Spectroscopy for Ethanol Characterization

**Figure 7:**
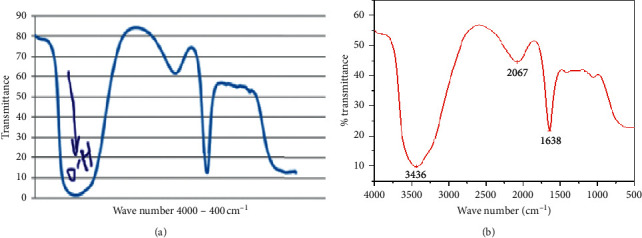
FTIR spectrum of the ethanol yield at acid concentration of 4% and temperature of 150°C and for a hydrolysis of 90 minutes. (a) Chiquere variety; (b) Gebabe variety.

When a liquid film runs between 4000 and 400 cm^−1^ regions with a varying intense and broad band, it indicated the O-H stretch of alcohols which was found between 3500 cm^−1^ and 3200 cm^−1^. The bands at around 1050 cm^−1^ and 2067 cm^−1^ were assigned as the symmetric stretching modes of the –CH_2_ and–CH_3_ groups, respectively. This ascertains that the product obtained from stalk residues of Chiquere and Gebabe varieties was ethanol, due to the presence of ethanol peaks (Figure 7).

**Figure 8:**
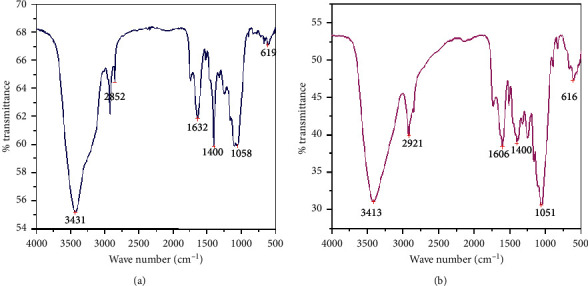
FTIR spectrum of the ethanol yield for pretreated acid of stalk residues of sweet sorghum. (a) Chiquere variety; (b) Gebabe variety.

Figure 8 shows FT-IR spectrum analysis of Chiquere and Gebabe varieties pretreated powder found between 3000 cm^−1^ and 1000 cm^−1^ is stretched band. The O-H stretch at 3431 cm^−1^, C-H stretch at 2852 cm^−1^, C-C peak at 1400 cm^−1^, and C-O vibration at 1632 cm^−1^ stretch for Chiquere variety and O-H condensed at 3413 cm^−1^, C-H elevation at 2921 cm^−1^, C-C peak at 1606 cm^−1^ and C-O vibration at 1058 cm^−1^ for Gebabe variety representing the standard ethanol (Figure 8). This confirmed that the cellulose and lignin content was removed through pretreating and hydrolysis with diluted sulfuric acid.

**Figure 9:**
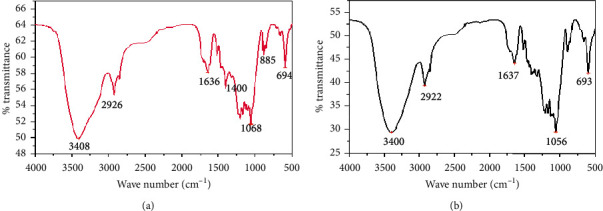
FTIR spectrum of the ethanol yield for untreated acid of stalk residues of sweet sorghum. (a) Chiquere variety; (b) Gebabe variety.

Figure 9 represents FTIR spectrum analysis of untreated stalks of Chiquere and Gebabe varieties. The O-H stretch at 3408 cm^−1^, C-H peak at 2926 cm^−1^, C-C vibration at 1636 cm^−1,^ and C-O condensed at 1058 cm^−1^ for Chiquere and O-H stretch at 3400 cm^−1^, C-H peak at 2922 cm^−1^, C-C elevation at 1637 cm^−1,^ and C-O peak at 1056 cm^−1^ for Gebabe variety were found to be similar as that of the standard ethanol. However, the band between 1500 and 1000 cm^−1^ for Chiquere and 1750 to 1000 cm^−1^ for Gebabe was found to be more condensed among each other (Figure 9). This condensed band may indicate the presence of cellulose and lignin component in both stalks.

## 4. Discussion

As observed in Table 1, stalk residuals of Chiquere variety have lowered the moisture content (4.33%) as compared to stalk residuals of Gebabe variety (6.67%), while stalk residuals of Chiquere variety had high volatile matter (48%) in counterpart to stalk residual of Gebabe variety, 42.6%. The study's result is not similar to the study reported by Gopakumar [32] who reported the moisture contents and volatile matter were 8.72 and 80.72, respectively, in sorghum. However, the moisture content of stalk residuals of Chiquere and Gebabe varieties was found to be nearly approached according to the study of Gopakumar [32] study result, but the volatile matter of both varieties was less than that. This difference may occur probably due to the differences in species of varieties used and collection time of stalks maturity stage. The moisture content is a measure of the amount of water in the stalks. Moisture content analysis is used for the determination of proportionality of solid-to-liquid ratio in the pretreatment and hydrolysis method; with increasing moisture content, it affects the product quality. Again, moisture content affects the ability of conversion of disaccharide sugar into monosaccharide sugar, storage stability, processing behavior, and quality and appearance of sugar [33]. The analysis of total moisture is used to determine other properties such as volatile matter. The sample of stalk residuals of Gebabe variety contains higher moisture content than stalk residuals of Chiquere variety, and it needs more heat for moisture vaporization.

Stalk residuals of Chiquere variety of sweet sorghum powder contain high extractives (2%), lignin (22%), hemicellulose (3%), and cellulose (41.5%) when compared to stalk powder of Gebabe variety, as shown in Table 1. However, the results obtained from this study were similar to the study reported by Saini et al. [11], who noticed that sweet sorghum bagasse contains lignin (14–21%), hemicellulose (18–27%), and cellulose (34–45%), except hemicellulose content. Therefore, the determination of cellulose and hemicellulose can be applied to quantify the theoretical production of ethanol. Stalks of sweet sorghum contain soluble free sugars, namely, glucose and sucrose, and insoluble carbohydrates such as cellulose and hemicellulose [34]. However, *Saccharomyces cerevisiae* only converts glucose and sucrose into ethanol. Thus, *Saccharomyces cerevisiae* cannot convert cellulose and hemicellulose into ethanol directly.

In this study, both stalk residuals of Chiquere and Gebabe varieties, cellulose and lignin content, were found in the range of the study reported by Saini et al. [11]. However, hemicellulose content was greater than that of a reported result by this author. There was medium level of lignin content that was observed in this study for both variety powders when relatively compared with another substrate lignin content, for example, it is 18–25% in hard wood stem, 25–35% in soft wood steam, 30–40% in nut shells, and 10–35% in grasses [35]. The lower lignin content was easy for hydrolysis condition and may reduce formation of toxic chemicals which are aromatic, polyaromatic, phenolic, and aldehydic.

Reducing sugar concentrations of stalk residuals of both varieties were determined by using phenol sulfuric acid method. The highest reducing sugar was found in the stalk residuals of Chiquere variety, i.e., 18.3%, while, in Gebabe variety, it was 7% (Table 2). In this study, stalk residuals of Gebabe variety reducing sugar were not similar to the studies reported by Hunsigi et al. [36], who reported highest reducing sugars were observed in RSSV 138 (14.78%) and the least reducing sugars were observed in NSSV 260 (11.35%) varieties of sweet sorghum. The range obtained was slightly lower than the findings of Batoul [37]; Guden et al. [38] found a range of 9.19 to 18.47%. The highest reducing sugar of Chiquere variety was 18.3%, which is found in the range of the study reported by Sir Elkhatim [39], who found lowest and highest reducing sugar contents were 13.64 and 18.78%, respectively, in sweet sorghum. The total sugar content of sweet sorghum was varied with varieties and maturity stage. However, as the crop matured, the total sugar content decreased as a result of the transfer of accumulated photosynthatase from the stalks sap towards the developing grains [40].

Ethanol production can be affected by many parameters starting from sample preparation to distillation. The fermentation steps have a complex connection with independent variables. Three different inoculum sizes (5%, 10%, and 15%) were investigated to determine the effect of inoculum size on kinetic parameters of ethanol fermentation from stalk residues of Chiquere and Gebabe varieties of sweet sorghum. The highest ethanol yield after 72 hours of fermentation from stalk residues of Chiquere variety was 22.40, 21.5, and 19.87; on the other hand, it was 22.17%, 21.78, and 19.88 in Gebabe variety, respectively (Table 7). Therefore, 5% yeast inoculum size resulted in higher ethanol yield as compared to 10% and 15% inoculum sizes. Reduced ethanol yield with increasing yeast concentration resulted from increased biosynthesis of glycerol which is nonfermentable. Table 7 showed that the maximum amount of ethanol yield was obtained for both varieties at 5% inoculum size over constant temperature of 30°C after 72 hours of fermentation period. The results demonstrated that there is a decrease of ethanol yield from 5% to 15% inoculum size; this may cause a decrease of kinetic parameters for ethanol fermentation by *S. cerevisiae*. According to Lin et al. [41], the ethanol production increases by inoculum up to 4% during ethanol production from sweet sorghum stalks juice. Among 5%, 10%, and 15% inoculum sizes, 5% was selected to be the optimum inoculum size by comparing ethanol production rate, maximum growth rate, and produced ethanol in this study. Inoculum size does not have significant influence on final ethanol concentration; however, it significantly affects sugar consumption rate and ethanol productivity [42]. Moreover, it was reported that ethanol production was decreased with the increased inoculum size cell numbers from 5% to 15%. There was also statistically significant difference in mean production rate among the inoculum sizes (*P* < 0.05). Increased cell concentration within a certain range also reduces fermentation time considerably due to the rapid growth of cells in the fermentation media that immediately consumes feeded sugars producing ethanol.

The stalk residue of Chiquere variety of sweet sorghum solution had a higher pH value as compared to stalk residue of Gebabe variety of sweet sorghum solution after pretreatment and hydrolysis by diluted sulfuric acid; this might be due to the low acid content in stalk in Gebabe one. The optimal pH was determined by fermenting stalk residues of Chiquere and Gebabe variety solutions at 3, 4, and 5 pH levels at 180 rpm and at 30°C temperature (Table 7). Ethanol content after 72 hours of fermentation in stalk residue of Chiquere variety was 19.94%, 21%, and 20.92%, and for Gebabe variety, it was estimated as 19.76%, 21.71%, and 20.53% at 3, 4, and 5 pH levels, respectively. Therefore, at 4 pH level, higher ethanol yield was produced as compared to 3 and 5 pH for both varieties. The initial and dynamic changes of pH level during fermentation have an effect on optimum alcohol yield, since the activity of zymase enzyme produced by *S. cerevisiae* is pH dependent [37]. The pH of stalk residue of Chiquere variety result was not significant, whereas, in Gebabe variety, it was found to be significant. The optimum pH value was found to be 4.0 for both the varieties. This pH range was somewhat approached to the findings of Chen [43], where the maximum ethanol productivity was achieved at pH of 4.2 to 4.5. Again, this study was nearest to the finding of Lin et al. [41], who reported that several feedstocks had different optimum pH ranges, such as 2.8 to 3.4 for sugarcane juice and 4.0 to 4.5 for sucrose. According to Gebremariam [44], the ethanol concentration increased with increase in pH to a maximum concentration of 4.7% (v/v) at pH 4.5, beyond which it started to show a slight decreasing trend. According to these results, pH 4.5 provides optimal condition for ethanol production. Another studies by Geetha and Kumar [45] reported significant increase in ethanol yield from pH of 4.5–5.5, after which the levels did not increase much. This result was not in accordance with that of the above literature from wet coffee processing waste and sunflower head waste, respectively. In these studies, the optimum pH level was lower; this may come from adaptability of *S. cerevisiae*. The pH of sweet sorghum juice during fermentation could influence the rate of yeast growth, metabolism, and ethanol production [39]. In this experiment, production of ethanol was increased with increasing the level of pH from 3.0 to 4.0 after this sudden decrease. The results showed that production of ethanol depended on the initial and dynamic changes of pH level during fermentation which have an effect on optimum alcohol yield, since the activity of *S. cerevisiae* was affected by pH. In addition to this, during ethanol fermentation, yeast cells are negatively affected by various stress factors. These stress factors include extreme temperatures, pH, nutrient deficiency, contamination, and cell metabolic conditions such as high sugar content tolerance and ethanol tolerance. [46].

Water was added to the stalk residues of Chiquere and Gebabe variety solution of sweet sorghum in different ratios to assess the influence of dilution rate and thus initial sugar concentration on the ethanol yield. The experimental error associated with these experiments was at 95% confidence limit. The effect of varying dilution rate on the ethanol yield is shown in Table 7 constant at 30°C and for 72 hours of fermentation. Ethanol content after 72 hours of fermentation from stalk of Chiquere variety was found to be 21.46%, 20.11%, and 19.55%, while it was 21.78%, 21.29%, and 19.86% in Gebabe variety at 10 ml, 20 ml, and 30 ml dilution rates, respectively. Therefore, dilution rate of 10 ml produced higher ethanol yield as compared to 20 ml and 30 ml dilution rates in both varieties. The highest ethanol yield of 1 : 10 solid to liquid of H_2_SO_4_ solution was observed at a dilution of 10 ml. The lowest ethanol yield was observed at 30 ml dilution. According to Gaur [47], high sugar concentrations above 20% cause an increase in osmotic pressure and the increase of osmotic pressure has been related with the increase in glycerol yield. It is believed that a sugar concentration of approximately 16 to 18% causes osmotic pressure effects on the yeast during fermentation and the added stress in the cells yields lower ethanol concentrations. Yeast cells adapt to osmotic stress environments by producing glycerol. Table 7 showed that at high sugar concentration (undiluted) the ethanol yield was low as compared to 30 ml dilution rate with reduced sugar concentration. This result is in line with the study reported by Gaur [47].

Amount of ethanol produced by fermentation was estimated by potassium dichromate method. The highest ethanol concentration was observed at inoculum size 5%, pH at 4.0, and dilution rate at 10 ml for both varieties (Table 7). The maximum ethanol concentration in acid pretreated stalks was 63.96% and 23.96% at pH 4.0 levels from the stalks of Chiquere and Gebabe varieties of sweet sorghum, respectively. It was found that the amount of ethanol concentration was small, when compared to the study reported by Evans [48], where the highest ethanol concentration was 99.60 %, while the lowest was 98.22% for Madhura variety of sweet sorghum, respectively. However, Batoul [37] reported that after 72 hours of fermentation, the alcohol content was 12.75, 10.02, and 10.27% for 5, 6, and 7 pH levels, respectively. Another work reported by Alemayehu [49] noted the maximum ethanol production 7.46% was observed at pH 4.5 in acid pretreated from potato peel wastes.

The broad band indicated the O-H stretch of alcohols was found between 3500 cm^−1^ to 3200 cm^−1^^1^ while the region from 1260 cm^−1^ to 1050 cm^−1^ confirms the C–O stretch. The bands at around 1400 cm^−1^ were assigned as the symmetric stretching modes of the –CH_2_ and –CH_3_ groups for both varieties (Figure 7) [24]. This ascertains that the product obtained from powder of both varieties is definitely ethanol due to the confirmation of these regions (Figure 7) [43].

The acid pretreated Chiquere variety band was found between 1500 cm^−1^ and 1000 cm^−1,^ whereas untreated Gebabe variety band was found between 1750 cm^−1^ and 1000 cm^−1^. FTIR result showed that diluted pretreated acid has the ability to remove lignin, hemicellulose, and cellulose content as compared with pretreated stalk residues (Figure 8). A number of acids may be used, such as HCl, H_2_SO_4_, H_3_PO_4,_ and HNO_3_. Sulfuric acid is commonly used due to low cost, nonvolatileness, and affordable corrosion strength [50]. This may be one of the reasons why diluted sulfuric acid pretreatment was suitable to release fermentable sugar from these varieties' powder. It has been stated that during the acid pretreatment, the structure of lignin, hemicellulose, and cellulose content is disrupted, thus making the carbohydrates more accessible to enzymes [51]. In this chemical pretreatment, mainly employed chemical agent was sulfuric acid for enhancing hydrolysis and improving glucose recovery from cellulose because of the removal of hemicellulose or lignin. Moreover, diluted acid hydrolysis has been successfully developed for pretreatment for lignocellulosic material [28]. Another study carried out by Kumar et al. [52] observed that acid concentration in the dilute acid hydrolysis process is in the range of 2 to 5%. 4% diluted sulfuric acid was used in this study at high temperature (120–200°C) for 90 minutes to break the cellulose chains into glucose. Concentrated acids such as H_2_SO_4_ have been used to treat lignocellulosic material. Although they are powerful agents for cellulose hydrolysis, concentrated acids are toxic, corrosive, and hazardous and require reactors that are resistant to corrosion. Dilute acid hydrolysis is selected for the production of fermentable sugars via softer conditions than those in the case of concentrated acid. This process used dilute acid concentration (up to 0.5 to 5%) at a temperature in the range of 100 to 240°C. In temperatures between 110 and 140°C, hemicellulose is hydrolysed, while cellulose was hydrolysed at high temperature up to 240°C. Chen [43] reported that during prehydrolysis, the lignin-hemicellulose complex is broken down, facilitating the hemicellulose hydrolysis and the sugar production, mainly xylose, under relatively soft conditions. However, at increased temperature, xylose is broken down and undesirable byproducts are formulated [53].

## 5. Conclusions

Results of our study revealed that the three factors were found to be significant for ethanol yield. Very low yeast inoculum size and dilution factors had positive effect on the yield of ethanol, whereas very high dilution rate resulted in negative impact on ethanol yield. As such, productions of ethanol from the stalks of Chiquere and Gebabe varieties of sweet sorghum are feasible from the economic point of view. It was also evidenced that efficient ethanol production can be obtained from sweet sorghum. The production of ethanol from sweet sorghum is a significant finding that can constitute a valuable way of using derivative products from sweet sorghum at farm level. Based on these facts, compared to food crops, sweet sorghum stalks which are an agricultural waste can act as promising alternative feedstock for bioethanol production. The use of this waste as an alternative can also reduce the environmental impacts arising from dumping of the waste directly to the nearby rivers or burning them as waste generating wasteful CO_2_ for the environment. It can also contribute to the solution of fossil fuel replacement in developing countries such as Ethiopia. Considering the remarkable potential of ethanol that can be produced from sweet sorghum, further improvement is still needed for maximum results, especially in the fermentation processes.

## Figures and Tables

**Table 1:** Physical and chemical properties of Chiquere and Gebabe varieties of sweet sorghum.

Proximate analysis	Chiquere (%)	Gebabe (%)
*Physical properties*		
Moisture	4.33 ± 0.68	6.67 ± 0.57
Volatile matter	48 ± 0.43	42.67 ± 0.73

*Chemical properties*		
Extractives	2 ± 0.23	1.8 ± 0.32
Lignin	22 ± 0.78	20 ± 0.83
Hemicellulose	37 ± 0.37	34. ± 0.43
Cellulose	41.5 ± 0.87	39.2 ± 0.69

**Table 2:** Absorbance of glucose by Chiquere and Gebabe varieties of sweet sorghum by using UV-visible spectrophotometer.

Concentration of glucose (mg/ml)	Absorbance at 490 nm		Sugar concentration (%)	
	Chiquere variety	Gebabe variety	Chiquere variety	Gebabe variety
20	1.7	3.1	6.67	5.42
40	25.4	4	9.12	7.5
60	28.0	4.5	12.5	10.83
80	31.0	5.3	15.83	13.33
100	35.3	7	22.5	21.5

**Table 3:** Estimation of known ethanol concentration by using UV-vis spectrophotometer.

Ethanol concentration (mg/ml)	Absorbance at 600 nm
20	19.2
40	20.3
60	21.8
80	23.5
100	25.3

**Table 4:** Analysis of variance (ANOVA) of Chiquere and Gebabe variety ethanol yield of sweet sorghum.

Chiquere variety						Gebabe variety					
Sources	Sum of squares	Degree of freedom	Mean squares	*F* values	*P* value (*P* > *F*)	Sources	Sum of squares	Degree of freedom	Mean squares	*F* values	*P* value (*P* > *F*)
Model	7.81	9	0.87	73.59	<0.0001	Model	2.24	9	0.58	120.30	<0.0001

A, yeast inoculum size	0.42	1	0.42	35.90	<0.0001	A, yeast inoculum size	0.28	1	0.28	58.16	0.0001

B, pH	6.125*E* − 004	1	6.125*E* − 004	0.052	0.9285	B, pH	0.11	1	0.11	21.28	0.0023

C, dilution	3.42	1	3.42	290.02	<0.0001	C, dilution	3.92	1	3.92	810.64	<0.0001

AB	0.096	1	0.096	8.15	<0.0001	AB	0.048	1	0.048	10.01	0.0158

AC	0.79	1	0.79	67.19	<0.0001	AC	0.53	1	0.53	110.20	<0.0001

BC	0.038	1	0.038	3.23	0.0012	BC	0.010	1	0.010	2.07	0.1936

A^2^	0.44	1	0.44	37.43	<0.0001	A^2^	6.737*E* − 003	1	6.737*E* − 003	1.39	0.2764

B^2^	1.28	1	1.28	108.53	<0.0001	B^2^	0.024	1	0.024	4.90	0.0625

C^2^	1.02	1	1.02	86.19	<0.0001	C^2^	0.32	1	0.32	65.85	<0.0001

Residual	0.083	7	0.012	-	-	Residual	0.034	7	4.836*E* − 003	-	-

Lack of fit	0.075	3	0.025	12.42	0.07899	Lack of fit	5.85*E* − 003	3	1.950*E* − 003	0.28	0.8390

Pure error	8.000*E* − 003	4	2.00*E* − 003	-	-	Pure error	0.028	4	7.000*E* − 003	-	-

Cor total	7.89	16	-	-	-	Cor total	5.27	16	-	-	-

*E* = 10^*X*^, where *X* is the power of decimal number.

**Table 5:** Model adequacy measures of Chiquere and Gebabe variety ethanol yield of sweet sorghum.

Chiquere variety			
Standard deviation	0.11	R-squared	0.9895
Mean	20.73	Adj R-squared	0.9761
C. V. %	0.52	Pred R-squared	0.8473
Press	1.20	Adeq R-squared	26.3888

Gebabe variety			
Standard deviation	0.070	R-squared	0.9936
Mean	20.41	Adj R-squared	0.9853
C. V. %	0.34	Pred R-squared	0.9739
Press	0.14	Adeq R-squared	39.937

**Table 6:** Regression coefficients and the corresponding 95% CI high and low Chiquere and Gebabe variety ethanol yield of sweet sorghum.

Chiquere variety						Gebabe variety					
Factor	Coefficient estimate	Degree of freedom	Standard error	95% CI low	95% CI high	Factor	Coefficient estimate	Degree of freedom	Standard error	95% CI low	95% CI high
Intercept	21.37	1	0.049	21.26	21.48	Intercept	20.33	1	0.031	20.26	20.40
A, yeast inoculum size	0.23	1	0.038	0.14	0.32	A, yeast inoculum size	0.19	1	0.025	0.13	0.25
B, pH	8.750*E* − 003	1	0.038	0.082	0.100	B, pH	−0.12	1	0.025	−0.17	−0.057
C, dilution	−0.65	1	0.038	−0.74	−0.56	C, dilution	−0.70	1	0.025	−0.76	−0.64
AB	0.16	1	0.054	0.027	0.028	AB	0.11	1	0.035	−0.19	−0.028
AC	0.44	1	0.054	0.32	0.57	AC	0.36	1	0.035	0.28	0.45
BC	−0.098	1	0.054	−0.23	0.031	BC	0.050	1	0.035	−0.032	0.13
A^2^	−0.32	1	0.053	−0.45	−0.20	A^2^	−0.040	1	0.034	−0.12	0.13
B^2^	−0.55	1	0.053	−0.68	−0.43	B^2^	0.075	1	0.034	−0.16	5.135*E* − 003
C^2^	−0.49	1	0.053	0.62	−0.37	C^2^	0.27	1	0.034	0.19	0.36

*E* = 10^*X*^, where *X* is the power of decimal number.

**Table 7:** Effect of various factors on ethanol production from Chiquere and Gebabe varieties of sweet sorghum.

Factors		Ethanol concentration (%)	
		Chiquere variety	Gebabe variety
Inoculum size (%)	Control (+ve)	21.69 ± 0.58	21.59 ± 0.59
	Control (−ve)	0	0
	5	22.40 ± 0.83	22.17 ± 0.78
	10	21.57 ± 0.42	21.7 ± 0.33
	15	19.8 ± 0.76	19.88 ± 0.18
pH	Control (+ve)	20.65 ± 0.62	20.53 ± 0.60
	Control (−ve)	0	0
	3	19.9 ± 0.43	19.76 ± 0.12
	4	21 ± 0.10	21.71 ± 0.27
	5	20.92 ± 0.25	20.53 ± 0.38
Dilution rate (ml)	Control (+ve)	20.57 ± 0.47	20.99 ± 0.89
	Control (−ve)	0	0
	10	21.46 ± 0.14	21.78 ± 0.33
	20	20.11 ± 0.15	21.29 ± 0.18
	30	19.55 ± 0.41	19.86 ± 0.10

*∗*Values are the mean of triplicates. ± indicates standard deviation.

## Data Availability

Raw data can be obtained from the corresponding author upon request. . .

## References

[1] Balat M., Balat H., Öz C. (2008). Progress in bioethanol processing. *Progress in Energy and Combustion Science*.

[2] Raghavendra B., Havannavar S. G. (2007). Pre-treatment of agroresidues for release of maximum reducing sugar. *Karnataka Journal of Agricultural Sciences*.

[3] Mohd Azhar S. H., Abdulla R., Jambo S. A. (2017). Yeasts in sustainable bioethanol production: a review. *Biochemistry and Biophysics Reports*.

[4] Nisha K. R., Noufal N. (2017). Bioethanol production from biomasses of fresh water Blooms-*Spirogyra* spp and *Eichhorniac rassipes* by *Saccharomyces cerevisiae*. *European Journal of Pharmaceutical and Medical Research*.

[5] Demirbas A. (2004). Combustion characteristics of different biomass fuels. *Progress in Energy and Combustion Science*.

[6] Ismaila U., Gana A., Tswanya N., Dogara D. (2010). Cereals production in Nigeria. Problems, constraints and opportunities for betterment. *African Journal of Agricultural Research*.

[7] Raud M., Tutt M., Olt J., Kikas T. (2015). Effect of lignin content of lignocellulosic material on hydrolysis efficiency. *Agronomy Research*.

[8] Akpan I., Uraih N., Obuekuwe K. M. (2004). Physi-cochemical characteristics of *Mahihot esculenta* plant. *Acta Biotechnologica*.

[9] Afolayan S. O., Ogedengbe K., Saleh A. (2012). Emission Stabilization using empirical evaluation of some agricultural residues as potential alternative energy and soil amendment sources. *International Journal of Advanced Scientific and technical Research*.

[10] Nasidi M., Akunna J., Deeni Y., Blackwood D., Walker G. (2010). Bioethanol in Nigeria: comparative analysis of sugarcane and sweet sorghum as feedstock sources. *Energy & Environmental Science*.

[11] Saini J. K., Saini R., Tewari L. (2015). Lignocellulosic agriculture wastes as biomass feedstocks for second-generation bioethanol production: concepts and recent developments. *3 Biotech*.

[12] Mupondwa E., Li X., Tabil L., Sokhansanj S., Adapa P. (2017). Status of Canada’s lignocellulosic ethanol: Part I: pretreatment technologies. *Renewable and Sustainable Energy Reviews*.

[13] Barbanti L., Grandi S., Vecchi A., Venturi G. (2006). Sweet and fibre sorghum (*Sorghum bicolor* (L.) Moench), energy crops in the frame of environmental protection from excessive nitrogen loads. *European Journal of Agronomy*.

[14] Chavan U. D., Patil J. V., Shinde M. S. (2009). An assessment of sweet sorghum cultivars for ethanol production. *Sugar Technology*.

[15] Maiti R., Satya P. (2014). Research advances in major cereal crops for adaptation to abiotic stresses. *GM Crops & Food*.

[16] Thabsile V. B. (2003). *Strategies to Improve Yield and Quality of Sweet Sorghum as Cash Crop for Small-Scale Farmers in Botswana*.

[17] Almodares A., Hadi M. R. (2009). Production of bioethanol from sweet sorghum: a review. *African Journal of Agricultural Research*.

[18] Blummel M., Rao S. S., Palaniswani A., Sha L., Reddy B. V. S. (2009). Evaluation of sweet sorghum (*Sorghum bicolor* L. Moench) used for bio-ethanol production in the context of optimising whole plant utilisation. *Animal Nutrition and Feed Technology*.

[19] ORSG (Oromia Regional State Government) (2012). *Socio Economic Profile of West Arsi Zone,*.

[20] Harmsen P., Huijgen W. (2010). *Literature Review of Physical and Chemical Pretreatment Processes for Lignocellulosic Biomass. Report number: ECN-E-10-013*.

[21] Chen H. (2014). Brief introduction to the biotechnology of lignocellulose. *Biotechnology of Lignocellulose: Theory and Practice*.

[22] Armstrong E., Clayton J., Deen M., Polk R., Hjalmerson E. (2018). Yeast fermentation: the effect of sugar type on ethanol production. *Journal of Under Graduate Biology Laboratory Investigations*.

[23] Sluiter A., Ruiz R., Scarlata C. (2008). *Determination of Structural Carbohydrates and Lignin in Biomass: Laboratory Analytical Procedure*.

[24] Yu Y., Lin K., Zhou X., Wang H., Liu S., Ma X. (2007). New C−H stretching vibrational spectral features in the Raman spectra of gaseous and liquid ethanol. *The Journal of Physical Chemistry C*.

[25] Albalasmeh A. A., Berhe A. A., Ghezzehei T. A. (2013). A new method for rapid determination of carbohydrate and total carbon concentrations using UV spectrophotometry. *Carbohydrate Polymers*.

[26] Alvira P., Tomás-Pejó E., Ballesteros M., Negro M. J. (2010). Pretreatment technologies for an efficient bioethanol production process based on enzymatic hydrolysis: a review. *Bioresource Technology*.

[27] Zhang J., Ma X., Yu J., Zhang X., Tan T. (2011). The effects of four different pretreatments on enzymatic hydrolysis of sweet sorghum bagasse. *Bioresource Technology*.

[28] Heredia-Olea E., Pérez-Carrillo E., Serna-Saldívar S. O. (2013). Production of ethanol from sweet sorghum bagasse pretreated with different chemical and physical processes and saccharified with fiber degrading enzymes. *Bioresource Technology*.

[29] Donghai W., Shawn W. U. (2010). Sorghum as dry land feedstock for fuel ethanol in Ethiopia. *Fuel Ethanol from Sorghum*.

[30] Kundiyana D. K., Bellmer D. D., Huhnke R. L., Wilkins M. W., Claypool P. L. (2010). Kernel grain sorghum. *Bioresource Technology*.

[31] Tgarguifa A., Abderafi S., Bounahmidi T. (2017). Modeling and optimization of distillation to produce bioethanol. *Energy Procedia*.

[32] Gopakumar S. T. (2012). *Bio-oil Production through Fast Pyrolysis and Upgrading to “Green” Transportation Fuels*.

[33] Nimbkar N., Kolekar N. M., Akade J. H., Rajvanshi A. K. (2006). *Syrup Production from Sweet Sorghum*.

[34] Mussatto S. I., Teixeira J. A. (2010). Lignocellulose as raw material in fermentation processes. *Current Research, Technology and Education Topics in Applied Microbiology and Microbial Biotechnology*.

[35] Sun Y., Cheng J. (2002). Hydrolysis of lignocellulosic materials for ethanol production: a review. *Bioresource Technology*.

[36] Hunsigi G., Yekkeli N. R., Kongawad B. Y. (2010). Sweet stalk sorghum: an alternative sugar crop for ethanol production. *Sugar Technology*.

[37] Batoul Y. H. A. (2006). *Evaluation of Sweet Sorghum Genotypes for Ethanol Production*.

[38] Guden B., Erdurmus C., Erdal S., Uzun B. (2020). Evaluation of sweet sorghum genotypes for bioethanol yield and related traits. *Biofuels, Bioproducts and Biorefining*.

[39] Sir Elkhatim A. F. (2003). *Ethanol Production by Yeast Fermentation of Sweet Sorghum Juice*.

[40] Almodares A., Hadi M. R., Ahmadpour H. (2008). Sorghum stems yield and soluble cabohdrates under phonologica stages and salinity levels. *African Journal of Biotechnology*.

[41] Lin Y., Zhang W., Li C., Sakakibara K., Tanaka S., Kong H. (2012). Factors affecting ethanol fermentation using *Saccharomyces cerevisiae* BY4742. *Biomass and Bioenergy*.

[42] Jaisil P., Laopaiboon L., Thanonkeo P., Laopaiboon P. (2007). Ethanol production from sweet sorghum juice in batch and fed-batch fermentations by *Saccharomyces cerevisiae*. *World Journal of Microbiology and Biotechnology volume*.

[43] Chen H. (2015). Lignocellulose Biorefinery Engineering: Principles and Applications. Elsevier Science. *Woodhead Publishing*.

[44] Gebremariam M. (2016). *Production of Bio-Ethanol from Corncob*.

[45] Geetha S., Kumar A. (2013). Ethanol production from degrained sunflower head waste by *Zymomonas mobilis* and *Saccharomyces cervisiae*. *International Journal of Agricultural Science and Research*.

[46] Bai F. W., Anderson W. A., Moo-Young M. (2008). Ethanol fermentation technologies from sugar and starch feedstocks. *Biotechnology Advances*.

[47] Gaur K. (2006). *Process Optimization for the Production of Ethanol via Fermentation*.

[48] Evans M. M. (2013). *The Potential of Sweet Sorghum [Sorghum Bicolor (L.) Moench] as a Bio- Resource for Syrup and Ethanol Production*.

[49] Alemayehu G. (2015). *Effect of pH and Substrate Concentration on Ethanol Production from Potato Peel Waste Using Saccharomyces cerevisiae*.

[50] Gladyshko Y. (2011). *Extraction of Hemicelluloses by Acid Catalyzed Hydrolysis*.

[51] Sumphanwanich J., Leepipatpiboon N., Srinorakutara T., Akaracharanya A. (2008). Evaluation of dilute-acid pretreated bagasse, corn cob and rice straw for ethanol fermentation by *Saccharomyces cerevisiae*. *Annals of Microbiology*.

[52] Kumar P., Barrett D. M., Delwiche M. J., Stroeve P. (2009). Methods for pretreatment of lignocellulosic biomass for efficient hydrolysis and biofuel production. *Industrial & Engineering Chemistry Research*.

[53] Yang S. T. (2007). Extractive fermentation for the production of carboxylic acids. *in Bioprocessing for Value-Added Products from Renewable Resources: New Technologies and Applications*.

